# Supplementary material for: Recent advances in cryotolerance biomarkers for semen preservation in frozen form–A systematic review
Source: PLoS One. 2024 May 22;19(5):e0303567. doi: 10.1371/journal.pone.0303567 (PMC11111053; doi:10.1371/journal.pone.0303567)
Supplement: Attachment — Submitted filename: Rebuttal-Report.docx [file pone.0303567.s002.docx]

Manuscript # PONE-D-23-40697

Entitled “RECENT ADVANCES IN CRYOTOLERANCE BIOMARKERS FOR SEMEN PRESERVATION IN FROZEN FORM – A SYSTEMATIC REVIEW”

In the revised version of the article, all the addressed points raised by the Editor/Reviewers are in red colored font.

| **S. No.** | **Points Raised** | **How dealt** |
| --- | --- | --- |
| 1. | When submitting your revision, we need you to address these additional requirements.  Please ensure that your manuscript meets PLOS ONE's style requirements, including those for file naming. The PLOS ONE style templates can be found at  https://journals.plos.org/plosone/s/file?id=wjVg/PLOSOne_formatting_sample_main_body.pdf and  https://journals.plos.org/plosone/s/file?id=ba62/PLOSOne_formatting_sample_title_authors_affiliations.pdf. | We have tried to follow the given format. |
| 2. | Please identify your study as "systematic review" in the title of your manuscript. | Added |
| 3. | Please provide a complete Data Availability Statement in the submission form, ensuring you include all necessary access information or a reason for why you are unable to make your data freely accessible. If your research concerns only data provided within your submission, please write "All data are in the manuscript and/or supporting information files" as your Data Availability Statement. | All data are in the manuscript and/or supporting information files |
| 4. | We note that Figure 3 in your submission contain copyrighted images. All PLOS content is published under the Creative Commons Attribution License (CC BY 4.0), which means that the manuscript, images, and Supporting Information files will be freely available online, and any third party is permitted to access, download, copy, distribute, and use these materials in any way, even commercially, with proper attribution. For more information, see our copyright guidelines: <http://journals.plos.org/plosone/s/licenses-and-copyright>.  We require you to either (1) present written permission from the copyright holder to publish these figures specifically under the CC BY 4.0 license, or (2) remove the figures from your submission:   a. You may seek permission from the original copyright holder of Figure 3 to publish the content specifically under the CC BY 4.0 license.    We recommend that you contact the original copyright holder with the Content Permission Form (http://journals.plos.org/plosone/s/file?id=7c09/content-permission-form.pdf) and the following text: “I request permission for the open-access journal PLOS ONE to publish XXX under the Creative Commons Attribution License (CCAL) CC BY 4.0 (http://creativecommons.org/licenses/by/4.0/). Please be aware that this license allows unrestricted use and distribution, even commercially, by third parties. Please reply and provide explicit written permission to publish XXX under a CC BY license and complete the attached form.”   Please upload the completed Content Permission Form or other proof of granted permissions as an ""Other"" file with your submission.  In the figure caption of the copyrighted figure, please include the following text: “Reprinted from [ref] under a CC BY license, with permission from [name of publisher], original copyright [original copyright year].”   b. If you are unable to obtain permission from the original copyright holder to publish these figures under the CC BY 4.0 license or if the copyright holder’s requirements are incompatible with the CC BY 4.0 license, please either i) remove the figure or ii) supply a replacement figure that complies with the CC BY 4.0 license. Please check copyright information on all replacement figures and update the figure caption with source information. If applicable, please specify in the figure caption text when a figure is similar but not identical to the original image and is therefore for illustrative purposes only. | Fig. 3 deleted. Its contents explained in the text. |
| 5. | Please include captions for your Supporting Information files at the end of your manuscript, and update any in-text citations to match accordingly. Please see our Supporting Information guidelines for more information: http://journals.plos.org/plosone/s/supporting-information. | Added |
| Reviewers' comments | | |
| 1. | Is the manuscript technically sound, and do the data support the conclusions?  Reviewer #1: Yes  Reviewer #2: Yes | Nothing to address |
| 2. | Has the statistical analysis been performed appropriately and rigorously?  Reviewer #1: N/A  Reviewer #2: N/A | Nothing to address |
| 3. | Have the authors made all data underlying the findings in their manuscript fully available?  The [PLOS Data policy](http://www.plosone.org/static/policies.action#sharing) requires authors to make all data underlying the findings described in their manuscript fully available without restriction, with rare exception (please refer to the Data Availability Statement in the manuscript PDF file). The data should be provided as part of the manuscript or its supporting information, or deposited to a public repository. For example, in addition to summary statistics, the data points behind means, medians and variance measures should be available. If there are restrictions on publicly sharing data—e.g. participant privacy or use of data from a third party—those must be specified.  Reviewer #1: No  Reviewer #2: Yes | Now provided. |
| 4. | 4. Is the manuscript presented in an intelligible fashion and written in standard English? Reviewer #1: Yes  Reviewer #2: Yes | In spite of nothing to address, however, we have improved it further. |
| 5. | Reviewer #1: This literature review explores a compelling subject related to semen cryotolerance biomarkers, offering a substantial amount of information. Upon examination, a notable gap in the existing body of research becomes apparent, particularly in the examination of metabolites previously identified as biomarkers. Notably absent are contributions from the referenced research group, and the absence of an opinion based on the research team's experience is evident, presenting an opportunity to enhance the text through the inclusion of such insights. This additional layer of analysis could significantly enrich the overall depth and scholarly value of the review. | Now have tried to conclude/include our experience on various topics discussed in the article. |
| 6. | Reviewer #2: This manuscript represents a comprehensive overview of studies that have addressed a topic that is highly relevant to the field of animal andrology - molecular markers of cryotolerance or resistance in semen. The search strategy and data evaluation is clearly presented, and the collected findings are well-presented. I do have several comments that could be considered by the authors: - Please, modify the section on the search strategy (lines 84-90). It is not necessary to repeat that the search/analysis included paper published until March 2023. | Repetition deleted. |
| 7. | - Several sections of the manuscript (specifically paragraphs regarding the fatty acids, aquaporins, HSP-2, CRISP-3, beta-HEX) resemble the review published by Duracka et al. (https://doi.org/10.3390/ijms24043379). Please, add or modify these sections to distinguish your information from the above-mentioned paper. | All sections have been modified. |
| 8. | - Please, add the animal species each referenced study was focused on, where missing. | Added. |
| 9. | - The section on improvements to PFE cryopreservation is very vague. There are no references, and overall, this section does not provide much insight. Either delete it or it should be extended to include different strategies, either physico- chemical or much more additives that could be used (not just extracts). | We have tried to strengthen this aspect. |
| 10. | - Aquaporins seem to hold a specific position in the manuscript. Technically, they are sperm markers, yet is there a reason as to why they deserve a separate section? | Deleted. |
